# Dissociation between Selecting and Orienting Attentional Reading Deficits: A Study in Adults with Epilepsy

**DOI:** 10.3390/brainsci14030252

**Published:** 2024-03-05

**Authors:** Eric Siéroff, Yael Slama, Jordane Manouvrier, Agathe Laurent

**Affiliations:** 1 LMC2—Laboratoire Mémoire, Cerveau & Cognition UR 7536, Institute of Psychology, Paris-Cité University, 71 Avenue Edouard Vaillant, 92100 Boulogne-Billancourt, France; 2 Maison de Santé Pluridisciplinaire, 95350 Saint-Brice-sous-Forêt, France; slama.yael@yahoo.fr; 3 Assistance Publique–Hôpitaux Universitaires de Marseille, 13005 Marseille, France; 4 Service de Neurochirurgie, Hôpital Lariboisière, 75010 Paris, France; agathe.laurent@aphp.fr

**Keywords:** attention, reading deficit, epilepsy, hemispheric lesion, visual field

## Abstract

Word reading requires a range of spatial attention processes, such as orienting to a specific word and selecting it while ignoring other words. This study investigated whether deficits of these spatial attention processes can show dissociations after hemispheric lesions. Thirty-nine patients with left or right focal epilepsy and 66 healthy participants had to read aloud four-letter words presented in the left and right visual hemifields. There were three successive blocks of presentation: in the unilateral block, a single word was presented in one of the visual hemifields; in the bilateral block, two words were presented simultaneously, one in each visual hemifield; in the cued block, two words were also presented, but only the cued word had to be reported. Twenty-one patients, twelve with a left and nine with a right hemisphere lesion, showed a word reading deficit. Four had specific difficulties in the cued block, suggesting an attentional selection reading deficit. Twelve patients had an asymmetric reading deficit, suggesting an attention orientation or a visual field deficit. Five patients had more complex deficits. The visual field presentation procedure may help to reveal different types of reading disorders in patients with epilepsy and to dissociate orienting and selecting deficits.

## 1. Introduction

When reading several words in a text, one must identify one word at a time, following an identification sequence [1] (but see [2], for another view). An attentional window of processing must be aligned with the outlines of each word so that the word identification system can selectively process the letters composing the word as a whole, and only those letters [3,4], even though readers obtain useful information from the next parafoveally visible word in the text [5]. A filter must control the delivery of just the letters from the location of the target word to the word identification system. Once a word has been identified, attention must shift to the location of the next visible word. This identification sequence requires an attentional window of processing to be oriented successively on each individual word [6]. Accordingly, the positioning of an attentional window of processing may require two different spatial attention processes in reading: selecting and orienting [7]. Selection is the exact alignment of the attentional window of processing with the target word while other words are filtered out or ignored. Orienting is the shift of the attentional window of processing toward a specific location, usually according to some routine depending on script direction. Consequently, two types of spatial attention deficits after a brain lesion are likely to affect reading. Deficits in selection or filtering should make it difficult to process a word flanked by other words, whatever their location on the left or on the right side. Deficits in orienting processes would produce asymmetric reading performance, left hemisphere lesions producing right-sided deficits, and right hemisphere lesions producing left-sided deficits. The goal of this research is to investigate whether deficits of these spatial attention processes can show double dissociation in patients after lesions or dysfunctions in the left or right hemispheres.

Several reading deficits are characterized by problems in spatial attention processes, grouped in the term ‘spatial dyslexia’ used by Siéroff [8]. Two of them are of special interest here, attentional dyslexia and neglect dyslexia. One of these deficits, namely attentional dyslexia, seems to specifically affect the attentional control required to select individual words or letters, sometimes independently of the visual hemifield. For example, Shallice and Warrington [9] described two patients, FM and PT with left temporo-parietal lesions, who had difficulties identifying words in a two-word display, committing migration or intrusion errors between the two words; for example, “win fed” was reported as “fin fed”. They also had problems detecting or identifying individual letters within a single word although they could read single letters and even single words. Thus, patients with attentional dyslexia may have a deficit, not necessarily specific to letters, that affects the identification of multiple items of the same type without a deficit in the identification of single items [10]. FM and PT did not have left-right asymmetry of reading errors, but FM presented a right homonymous hemianopia without macular sparing and right sensory inattention, and PT a right-sided homonymous hemianopia. Another patient, FL, described by Mayall and Humphreys [11], showed prevalent letter migrations from the word presented on the right into response to the word presented on the left, after multiple bilateral mainly temporo-parietal lesions caused by carbon monoxide poisoning. He also showed possible signs of mild difficulties on the left side in reading. The patient PF, described by Siéroff [12], had difficulties reading short words, when presented tachistoscopically in a two-word bilateral display, with one word in each visual hemifield (43% correct responses), after a left parietal vascular lesion. He made some migration errors, but, most of the time, reported only one word and ignored the other, without strong asymmetry. His difficulties increased considerably in a cued partial report condition, in which a spatial cue indicated the single word to report in a two-word display. With the cue, his performance dropped to 9% correct, even though the task seemed simpler because he had to report only one word instead of two. His deficit might affect selecting one word when several words were presented, and the difficulty increased when the selection was forced by an explicit cue compared to when it was included in a routine schema involved in normal fluent reading. He did not present any asymmetry of reading errors and had no orienting deficit like spatial neglect. Thus, an attentional selection deficit might be independent of an orienting deficit.

Other spatial dyslexia can occur because the lesion produces a pathological bias affecting the orienting of attention, producing asymmetric performance. For example, in spatial neglect, a deficit in which patients ignore stimuli located on the contralesional side, patients make frequent errors reading words located in the contralesional space in a text or in a two-word bilateral display [13,14,15], or reading letters located in the contralesional part of a single word, mainly involving letter omission and substitution errors [16,17]. This type of deficit, namely neglect dyslexia, is frequent after lesions of the posterior part of the brain, and can sometimes occur without spatial neglect for nonverbal stimuli [18,19]. The asymmetric errors in text and in single-words can be dissociated [20], the first errors being sometimes related to an egocentric deficit, where the neglected side is defined with reference to the patients’ body, and the second to an allocentric or object-based deficit, defined with reference to the word spatial coordinates [19]. In the present study, we were particularly interested in egocentric deficits, because they show the importance of some orienting process to shift attention from one word to the other. Rich and Palmer [13] found that not only patients made word omission errors when two words, one in each visual hemifield, were presented and only one cued word had to be reported, but they sometimes replaced the target word by the distractor word located on the right ipsilesional side. These intrusion errors were interpreted by the authors as a mislocalization of the contralesional cue or of the contralesional word in the two-word pair. The question of the present study is whether the orienting deficits can be dissociated from the selecting deficit evoked in attentional dyslexia.

In reality, things are more complex because asymmetric performance in reading is not always explained by an attentional orienting deficit. An asymmetric reading deficit may also occur after lateral homonymous hemianopia, a complete loss of vision in one hemifield, or amblyopia, a visual deficit sparing some luminance sensitivity while impairing shape processing in parts of the visual hemifield [21,22]. Also, a lesion in the corpus callosum, specifically the posterior part or splenium, produces a disconnection between hemispheres and asymmetric reading performance, with difficulties reporting words presented in the visual hemifield ipsilateral to the language-dominant hemisphere [23].

Several methods have been used to detect and characterize spatial reading deficits. One of them, the divided visual field method corresponds to the presentation of words or letter strings in the left (LVH) and/or right (RVH) visual hemifields. It is a sensitive method, because the time necessary to extract features of each letter is limited, with a tachistoscopic presentation of the words usually of less than 200 ms [24,25]. This method allows to detect orienting deficits, with asymmetry between LVH and RVH words, and selecting deficits, with special difficulties in cued partial report condition, in which the selection is forced by an explicit cue [12]. Moreover, the method evaluates reading in each hemisphere, because each visual hemifield projects to the contralateral hemisphere, even though early callosal communication between hemispheres occurs [26].

In this study, we presented words in the LVH and/or RVH to patients with intractable epilepsy, with a left or right hemisphere epileptic focus. Focal epileptic seizures are characterized by brief motor, sensory, cognitive, autonomic, or psychic symptoms with or without altered consciousness. Most of the time, an acquired or developmental brain injury is the cause of the seizures. The cognitive consequences of epilepsy depend on the laterality and location of the epileptic focus, the type of lesion, the age of onset, the duration of the disease, the nature and frequency of seizures, and the type and number of anti-epileptic drugs taken, although the relationship between epilepsy and cognition is complex and bidirectional [27]. Surgical intervention for patients with refractory focal epilepsy may also have its own clinical consequences. Considering the present study, it is important to note that reading and attention deficits are frequently cited among the cognitive difficulties following intractable epilepsy. Several studies conducted in children have shown that the ability to learn to read is affected by epilepsy [28,29,30], and more than 40% of adult patients with intractable temporal epilepsy had some academic achievement deficiencies due to reading deficits [31]. Epilepsy can also affect different aspects of attention [32,33,34,35], and attention may be particularly important for the visual field method used here, which requires participants to focus and orient on lateralized stimuli, select one of two stimuli, and to sustain a high level of capacity with tachistoscopic presentations.

We used a tachistoscopic presentation of four-letter French words in three successive blocks of presentation in a reading-aloud task (Word experiment). In the unilateral block, only one word was presented in the LVH or RVH. In the bilateral block, two words were presented, one in the LVH and one in the RVH, and both had to be reported. In the cued block, two words, one in the LVH and one in the RVH, were presented with a simultaneous arrow cue, but only the cued word had to be reported. The blocks were presented in a fixed order, unilateral, then bilateral, then cued, for a direct comparison between patients. We also ran an experiment presenting object drawings stimuli with an object naming task (Object experiment) to evaluate the reading specificity of the deficits. Objects drawings were presented in the LVH and the RVH in three presentation blocks, unilateral, bilateral, and cued. However, not all the patients were able to take part in it and run both Object and Word experiments. 

We conducted analyses between groups of participants and predicted worse performance in patients than in controls. The tachistoscopic presentation of words should limit the extraction of visual information and affect the number of words correctly identified, depending on patients’ reading fluency. For example, limited presentation time affects neglect dyslexia, sometimes changing the pattern of errors within a single word [36]. 

In neuropsychology, group analyses are sometimes problematic because they can mask diverse and interesting cases. We therefore completed our analyses by comparing individual patients to control participants. We sorted patients into two reading deficit groups according to our hypotheses: a group with an attentional selection deficit and a group with an attentional orienting deficit. 

The comparison of the different presentation blocks may help to reveal attentional selection deficits. In the unilateral block, selection was simple because only one word was presented. In the bilateral two-word block, in which both words had to be identified and reported, selection was included in a routine schema; this is the schema involved in sentence reading (in scripts that are read from left to right), beginning with the leftmost word first, then with the following word on the right. In the cued block, two words were presented as in the bilateral block, but only the cued word had to be identified and reported (partial-report cue); a more appropriate schema had to be triggered and the routine reading schema had to be avoided. Thus, more attentional resources were required for selection and we predicted that attentional selection deficits should be aggravated in the cued block, compared to the other blocks. In addition, an analysis of letter processing in each position in the words provided information on the migration or intrusion errors in the case of a selection deficit. Intrusion errors are errors of commission, in which a letter from the non-target word is substituted for a letter in the target word. This kind of error can occur in healthy participants, most frequently in the LVH [37], but are more frequent in patients with attentional dyslexia [9]. 

Concerning the attentional orienting deficit, we hypothesized that the side of the lesion should affect the difference in accuracy between the LVH and RVH, left hemisphere orienting deficits producing asymmetry in favor of the LVH, and right hemisphere orienting deficits producing asymmetry in favor of the RVH. Our hypothesis was that orienting deficits might be dissociated from selection deficits. However, note that an RVH advantage has been described in normals when only a single word must be identified, in both unilateral [38] and cued conditions [39]. Conversely, in bilateral conditions, the report bias may favor the LVH word, or at least reduce the RVH advantage, due to preferences for left-to-right order of report or scanning [40]. These normal asymmetries have to be taken into account when considering asymmetries found in patients. Additionally, we have seen that asymmetry can be caused by other types of deficits, like hemianopia and callosal disconnection, and these possibilities will be individually discussed. 

## 2. Method

### 2.1. Participants

Thirty-nine patients with drug-resistant focal epilepsy, 21 with a left-sided and 18 with a right-sided epileptic focus, performed the Word experiment (21 women, 18 men; mean age = 27.9, SD = 10.0; range: 15.6–50.1). One patient had a bilateral, mainly right-sided, epileptic focus and was included in the right-sided epileptic focus group. Patients were included in the study after consulting in the Epilepsy Unit of the Department of Neurosurgery at Sainte-Anne Hospital, Paris. They were candidates for surgery or had already been operated on when tested. Sixteen patients were seen after a unilateral resection, within a minimum period of two months, and 23 were seen before the resection. We also ran 66 control participants without epilepsy or any other neurological antecedents (38 women, 28 men; mean age = 28.9, SD = 9.0; range: 17.2–56.8). Participants were included if they were native French speakers, and had normal or corrected visual acuity. All patients had undergone an electroencephalography (EEG), anatomical and functional magnetic resonance imaging (MRI and fMRI), F-fluorodeoxyglucose positron emission tomography ([18F]-FDG-PET scan) and a neuropsychological assessment. The neuropsychological assessment included the Wechsler Adult Intelligence Scale, fourth edition (WAIS-IV; Wechsler [41]), and clinical evaluations of executive functions with EpiTrack [42], lexical access with Lexis [43] and episodic memory with 13 words and drawings adapted from Jones-Gotman et al. [44]. A standardized reading test, the Alouette [45], was also administered to patients. Handedness was evaluated with the Edinburgh Handedness Inventory [46]. Except for one ambidextrous and five left-handed patients, and three ambidextrous and eight left-handed controls, other participants were right-handed. Language dominance of patients was determined by the different brain imaging techniques performed in the Epilepsy Unit (see above). All participants gave their informed consent, and the experiment was conducted in accordance with the ethical standards of the University of Paris-Cité. 

### 2.2. Word Experiment

#### 2.2.1. Design

The 105 participants (39 patients, 66 controls) identified 90 words in a reading aloud task, in six conditions (3 × 2 design): three presentation blocks (unilateral, bilateral, cued) in two visual hemifields (LVH, RVH). Target words were presented in one-word displays in the unilateral block and in two-word displays in the bilateral and cued block. In the cued block, the target words were presented with 30 filler words, and an arrow cue was simultaneously presented.

#### 2.2.2. Stimuli

The stimuli were 120 French four-letter words. Eight lists of 15 words were created: six for target words in the six conditions (three presentation blocks × 2 visual hemifields), and two for filler words in the cued block. The eight lists consisted of four-letter monosyllabic words, matched for written frequency, from 3.59 to 3.77 per 100 million [47]; for the number of orthographic neighbors, from 66 to 88 per list, with 14 words per list having at least one orthographic neighbor [48]; for imageability, with a mean score per list between 1.93 and 2.40 on a subjective scale ranging from 1 to 3; and for shape, with a similar number of ascenders or descenders in the letters. All letters were presented in lowercase, using 30-point Courier font. The fixation item, a + sign, was presented in the middle of the screen, subtending a visual angle of 0.4°. Words covered a visual angle of 1.4° and were centered at 1.6° from the fixation item, so the distance between the fixation item and the nearest letter was 0.9°. Arrows in the cued block were “<” (for left) and “>” (for right) symbols in place of the fixation item and subtended 0.6° of visual angle. A patterned mask made of irregular lines covered the whole display with a visual angle of 7.2° × 3.2°. All stimuli were presented in black on a white video screen. Participants sat approximately 57 cm from the screen so that 1 cm on the screen corresponded to 1° of visual angle.

#### 2.2.3. Procedure

**Figure 1:**
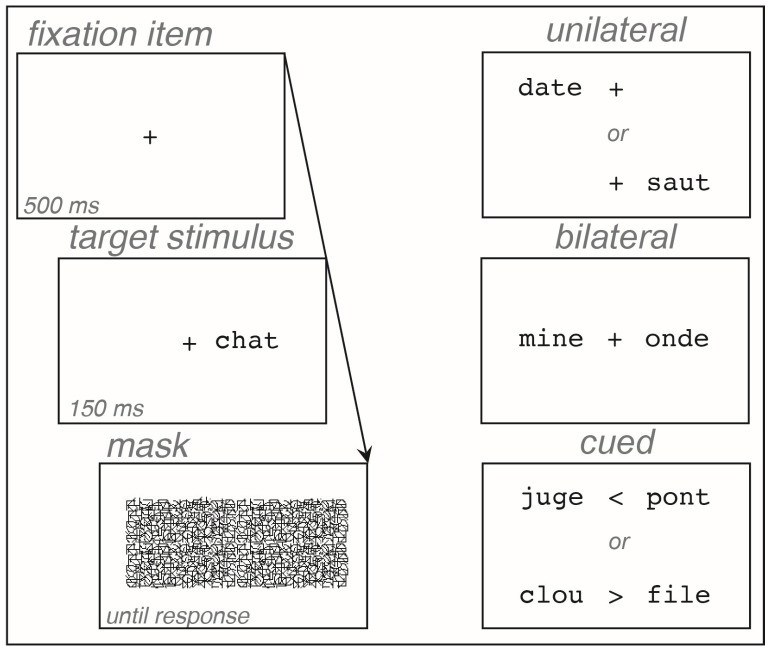
Sequence of stimuli in a trial, and different presentation blocks.

Control participants were tested at home or in the hospital, and patients were tested in the hospital in a quiet room or in their bedroom. The experiment was run on a MacBook Pro (Apple™), with a 17″ screen, using SuperLab™ 4.0. Participants responded to an information questionnaire, read the Alouette text, and then took the experiment. Each trial began with the presentation of the fixation item in the center of the screen for 500 ms, immediately followed by the word display for 150 ms, then by the patterned mask, which remained on-screen until the next trial. In the unilateral and bilateral blocks, the fixation item remained on-screen while the words were presented. In the cued block, a cue (arrow), indicating the target word to be reported, replaced the fixation item during the word display (Figure 1). Participants were required to look at the fixation item, not to move their eyes, and to identify and read aloud the target words, by either naming or spelling them orally. The experimenter transcribed each response and triggered a new trial immediately after the response was given. The experiment was composed of three successive blocks, corresponding to each presentation block, in a fixed order for a direct comparison between patients: unilateral, then bilateral, then cued. Each block was preceded by a short practice using different words from the experiment. In each presentation block, the word order was pseudorandomly determined and fixed for all participants. In the bilateral and cued blocks, words were associated so that they did not share more than one letter and did not have a strong semantic relationship. For each presentation block, the lists of words were counterbalanced so that each word was seen in LVH or RVH by an equal number of participants. The experiment lasted approximately 15 min.

### 2.3. Object Experiment

An experiment with drawings of objects was also administered, but only some of the participants were able to be included in this experiment: eight patients with a left hemisphere epileptic focus, seven patients with a right hemisphere focus, and 33 control participants. The procedure was the same as for the Word experiment, except that the presentation time of the stimuli was 100 ms. The stimuli were 90 target and 30 filler object drawings selected from Snodgrass and Vanderwart’s [49] standardized set. The drawing size was adapted so that maximum width and height of the stimuli were both 1.8° of visual angle. The task was to name the objects. Each block was preceded by a short practice using different drawings of objects from the experiment. The experiment was run after the Word experiment with a short pause between both experiments.

## 3. Results

### 3.1. Global Analyses

#### 3.1.1. Word Experiment

We measured the number of words for which participants pronounced the correct word or spelled all and only the correct letters of the word. A synonym or a word with one phoneme added was counted as incorrect. The word identification score of the 39 patients and the 66 control participants was entered into a repeated-measures ANOVA with Group (left-lesion patients, right-lesion patients, healthy controls) as a between-subject variable, and Visual hemifield (LVH, RVH) and Presentation block (unilateral, bilateral, cued) as within-subject factors. Table 1 shows the results. The maximum score per condition was 15. 

There was an effect of Group, *F*(2, 102) = 32.96; *p* < 0.001; η^2^ = 0.39. Post hoc analyses using Duncan multiple range tests revealed higher scores in healthy controls (*M* = 13.1, *SD* = 1.1) than in left- (*M* = 9.5, *SD* = 3.5) (*p* < 0.001) or right-lesion patients (*M* = 10.4, *SD* = 2.0) (*p* < 0.001), but no significant difference between the two groups of patients (*p* = 0.12).

**Figure 2:**
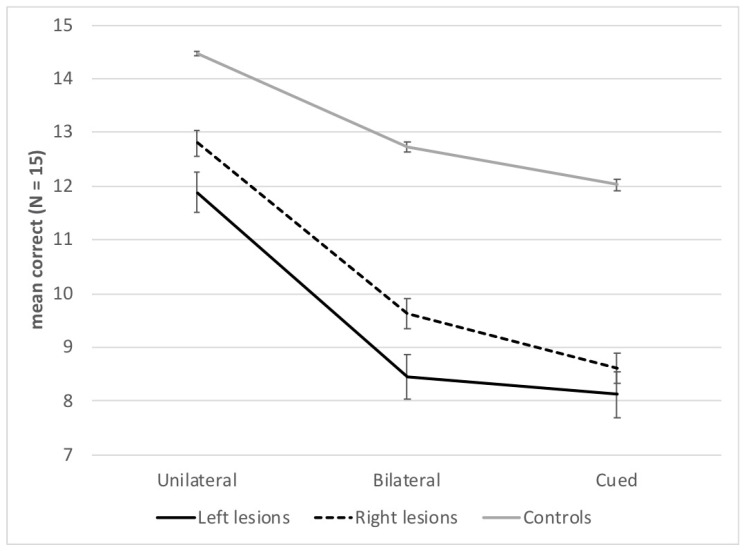
Mean number of correct words (*n* = 15) and standard errors for each group of participants and presentation block.

There was an effect of Presentation block, *F*(2, 204) = 201.14; *p* < 0.001; η^2^ = 0.66, with a gradation of performance between the three presentation blocks; scores were higher in the unilateral (*M* = 13.7, *SD* = 2.1) than the bilateral block (*M* = 11.3, *SD* = 2.9), and in both these blocks than in the cued block (*M* = 10.7, *SD* = 3.0) (*p*s < 0.001). The interaction between Group and Presentation block was also significant, *F*(4, 204) = 8.12; *p* < 0.001; η^2^ = 0.14. Both left- and right-lesion patients scored lower than healthy controls in all blocks (*p*s < 0.001), and patients with left lesions had lower scores than patients with right lesions in the unilateral and bilateral blocks (*p*s < 0.001), but not in the cued block (*p* = 0.14). Figure 2 also shows that the difference between patients’ and controls’ performance was larger in both bilateral and cued blocks than in the unilateral block.

There was no effect of Visual hemifield, *F*(1, 102) = 1.54; *p* = 0.22. However, there was a significant interaction between Presentation block and Visual hemifield, *F*(2, 204) = 78.46; *p* < 0.001; η^2^ = 0.43. In the unilateral and cued blocks, the score was higher for the RVH than the LVH, as expected when a single word has to be reported, but the score was higher for the LVH than in the RVH in the bilateral block, probably due to report bias (*p*s < 0.001). There was no interaction between Group and Visual hemifield, *F*(2, 102) = 0.41; *p* = 0.66, but there was a significant interaction between Group, Presentation block, and Visual hemifield, *F*(4, 204) = 4.75; *p* < 0.01; η^2^ = 0.09. The RVH advantage was found in the unilateral block only for the right-lesion patients (*p* < 0.001), and not for the controls (*p* = 0.27) and left-lesion patients (*p* = 0.13). Controls might show a ceiling effect in this block, explaining the lack of RVH advantage, but this is not the case for the left-lesion patients. An LVH advantage in the bilateral block and an RVH advantage in the cued block were found for all groups (*p*s < 0.001). Finally, patients’ scores were lower than controls’ scores in all conditions (*p*s < 0.001), except for right-lesion patients in the unilateral RVH condition (*p* = 0.13) (see Table 1). 

#### 3.1.2. Object Experiment

The object identification score of the 15 patients and the 33 control participants was entered into a repeated-measures ANOVA with similar factors to the Word experiment. There was an effect of Group, *F*(2, 45) = 10.22; *p* < 0.001; η^2^ = 0.31, with healthy controls scoring higher (*M* = 12.6, *SD* = 0.9) than left- (*M* = 10.6, *SD* = 2.8) or right-lesion patients (*M* = 10.2, *SD* = 2.2) (*ps* < 0.01), but no significant difference between the groups of patients (*p* = 0.51).

### 3.2. Subgroup Analyses

#### 3.2.1. Specific Scores

The global word score corresponded to the mean number of correct words in all conditions. Twenty-one patients had a global word score that was two standard deviations (SD) below the mean global word score of healthy controls, 12 out of the 21 patients with a left hemisphere lesion and 9 out of the 18 patients with a right hemisphere lesion. These patients are listed with their main characteristics in Table 2, and with their results in the Word and Object experiments in Table 3. Note that 13 of these 21 patients with a global word score below two SD were surgically treated, and only 3 of the other 19 patients were surgically treated.

We calculated selection and asymmetry specific scores for each participant in order to differentiate the two types of reading deficits with attentional processes deficits, and compared patients’ scores to those of the control participants, with a confidence level of two SD. The selection score was the mean difference between the unilateral and the cued blocks. In both blocks, only one word had to be reported, but selection was necessary in the cued block because of the partial report. A selection deficit should be specifically affected by the cued block. The asymmetry score was the mean difference between RVH and LVH. It was calculated independently of the presentation block because orienting is necessary in all blocks.

We then grouped patients with an abnormal global word score according to the most severe specific score, even though some patients may have had deficits affecting several scores. Because the patients’ deficits are complex, and in the absence of detailed case studies, the following classification is just an indication of the most frequent reading deficits and their possible explanations. We identified three subgroups, with a selection deficit, an abnormal asymmetry with contralesional deficit, or an abnormal asymmetry with ipsilesional deficit. Some patients without selection deficit or abnormal asymmetry were also listed in a fourth subgroup.

In a complementary analysis, we calculated the percentage of trials with migration errors among all trials with errors in the cued block. Migration errors were errors in which at least one or several letters in the response corresponded to one or several letters of the uncued word.

#### 3.2.2. Selection Deficit

**Figure 3:**
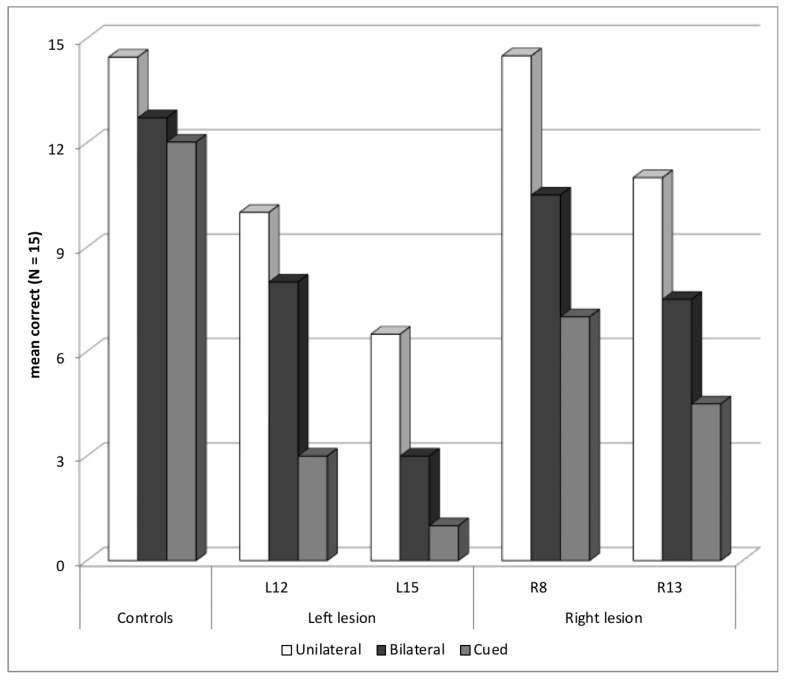
Patients with a Selection Deficit: Mean Correct Words for Each Presentation Block.

Four patients showed an abnormal selection *z*-score in the Word experiment, two with a left temporal or occipito-parietal lesion—L12 (selection *z*-score = −3.0) and L15 (−2.0)—and two with a right temporal lesion—R8 (−3.4) and R13 (−2.7). All patients also had some difficulties in the bilateral block, but the deficit was worse in the cued block, showing that their main difficulty may not consist in reporting more than one word (Figure 3). None of these patients presented an abnormal asymmetry score (max = −0.8), showing that the deficit was in selecting attention and was not related to orienting of attention.

Two patients, L15 and R8, were also tested with objects. L15 showed a global object score deficit (−3.5) and abnormal asymmetry for objects (−2.4), with a contralesional RVH deficit, but neither L15 or R8 showed a selection deficit for objects, suggesting that their selection deficit was word-selective. Another possibility is that performance improved in the Object experiment because of learning strategies, the Object experiment being performed after the Word experiment.

Three patients of this subgroup, L12, L15 and R13, had a global word score more than five SD below healthy controls, showing that the reading deficit was severe. Clinical characteristics showed that they also presented some language difficulties, with a lexical access deficit (*z*-score of −7.0, −13.3, and −5.1, in the Lexis, respectively). L12 and L15 had abnormal speed scores on the Alouette reading test without an accuracy deficit, but only L12 was known has having antecedent of dyslexia. L12 and L15 also had some executive function deficit.

Three patients of this subgroup showed more than 50% of migration errors in the cued block: L12 (54%), L15 (75%), and R13 (81%). We looked more closely at the types of migration errors made by patients L15 and R13, separating true migration errors in which one or several letters from the uncued words were included in the response along with other letters from the cued word, and another type of migration errors in which the response consisted entirely and only in the uncued word without letters from the cued words. We called intrusion the first type or errors, and transposition the second type of errors.

L15 suffered from a left lateral parieto-occipital ependymoma, resected at the age of one year. He made 28 errors in the 30 trials in the cued block, with migration errors in 21 trials (75%), 10 in the LVH and 11 in the RVH. Nineteen of his errors were true migration or intrusion errors, frequently associated with other types of errors (substitutions with another unrelated letter, omissions, and more rarely additions), but sometimes pure. For example, in the display boue > pain [mud, bread] in which pain had to be reported, his response was bain [bath] instead of pain with migration of the letter B in first position; in the display aide *<* port [help, port], his response was aire [area] instead of aide, with migration of the letter R in third position. 

R13 suffered from a right hemisphere cortical dysplasia near the middle and inferior temporal sulci. He made 21 errors in the 30 trials in the cued block, with migrations in 17 trials (81%), 6 in the LVH and 11 in the RVH. He made intrusion errors like L15, but he also made transposition errors in six trials, for example responding clou [nail] instead of file [lane or queue] in the display clou *>* file. Four of his 11 intrusion errors consisted of only one letter correct and three letters from the uncued word, and might be considered as mixed intrusion/transposition errors. So R13 not only had difficulties filtering out the uncued word, but he might have difficulties deciding which word to respond to. Possibly, several types of selection deficits exist.

#### 3.2.3. Abnormal Asymmetry: Contralesional Deficit

**Figure 4:**
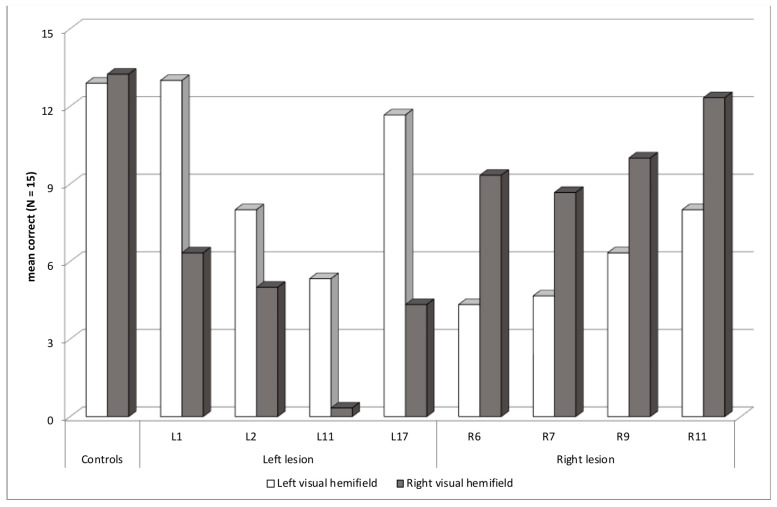
Patients with an asymmetric contralesional deficit: mean correct words in each visual hemifield.

Eight patients had an abnormal asymmetry score, at least two SD above or below the controls’ mean asymmetry score, in the Word experiment, consistent with a contralesional deficit (Figure 4). Four patients had a left lesion with an RVH deficit—L1 (asymmetry *z*-score = −5.4 SD), L2 (−2.6), L11 (−4.1) and L17 (−6.0), and four patients had a right lesion with an LVH deficit—R6 (+3.6), R7 (+2.8), R9 (+2.6), and R11 (+3.1). Seven patients had lesions involving the temporal lobe plus another lobe, occipital, parietal or frontal, and one patient had an insular lesion.

None of the four patients with left hemisphere lesions had truly detected clinical neglect, but L11 made contralesional right-sided omissions in the cancellation test. L1 and L17 had upper RVH quadranopia, but had acceptable RVH performance in the unilateral block of the Word experiment, showing that the visual field deficit was not the main cause of the abnormal asymmetry score in the Word experiment. Thus, these patients, along with L2, who did not show any visual field deficit, may present an attentional orienting deficit. L11 had a complete RVH hemianopia, which might explain the severe contralesional deficit obtained in the Word and Object experiments, and occurring in all three blocks. However, his low performance with words in the LVH, explaining a very low global identification score (−9.6), showed that he might present another deficit, specific to reading. 

No patient with a left lesion showed an abnormal selection score at least two SD below the mean selection score of healthy controls. However, two patients had a high percentage of migration errors, L1 (91%) and L11 (58%). L1 made 11 errors in the 30 trials in the cued block, with nine intrusions and one transposition, with a total of two migration errors in the LVH and eight in the RVH. Apart from one transposition error, her migration errors concerned only one letter. She may present a mixed orienting/selection deficit, but without specific difficulties in the cued block, in which performance was numerically higher than the bilateral block. The case of L11 was different, because he did not report any of the letters presented in the RVH cued words, certainly due to his right hemianopia. Most of his migration errors were transpositions, reporting an LVH word in six trials when an RVH word was cued, maybe because he reported the only word he saw, without a real attentional selection deficit. 

Concerning clinical characteristics, L11 and L17 had antecedents of dyslexia in childhood and were still abnormally slow on the Alouette test, with a non-negligible error rate. L11 had difficulties with lexical access (*z*-score of −4.0 in the Lexis) and an IQ at 69. Thus, reading and/or language difficulties were clinically assessed only in two of the four patients of this subgroup with left hemisphere lesions. Finally, L2 showed a clinical deficit of executive functions, and L1 some memory deficit.

The four patients with right hemisphere lesions had contralesional LVH deficit in the Word experiment only in the unilateral and cued blocks. In the bilateral block, their performance was better in the LVH than in the RVH. Patients may have ordered their responses by reporting the leftmost word first with the consequence of an absence of contralesional deficit as if it had been compensated by the report bias. These four patients may have an LVH attentional orienting deficit not severe enough to prevent left-report bias in the bilateral block. In the Object experiment, R6 had severe difficulties without asymmetry (global score −8.0), and R7 had no specific deficit. These patients may have an LVH attentional orienting deficit specific to reading. Another possibility is that performance improved in the Object experiment because of learning strategies, the Object experiment being performed after the Word experiment. 

R9 showed an abnormal selection score (−2.0). He made 65% of migration errors in the cued block, with nine intrusion errors in the LVH and two in the RVH. Possibly, he presented a mixed selection/orienting deficit, contrarily to the three other patients, in which the orienting deficit was not associated with a selection deficit.

Clinical characteristics showed that none of the four patients with right hemisphere lesions had truly detected clinical neglect, but R7 had a contralesional left-ear extinction in the dichotic test. R9 also showed a superior LVH quadranopia. However, his LVH performance in the unilateral and bilateral blocks of the Word experiment indicates that the visual field deficit cannot totally explain the asymmetry. Concerning other clinical characteristics, only R7 had antecedents of dyslexia in childhood. He also had difficulties with lexical access (*z*-score of −2.6 in the Lexis) and had a verbal memory deficit. R9 was abnormally slow on the Alouette test. He also had difficulties with lexical access (*z*-score of −10.0 in the Lexis) and a low verbal comprehension index in WAIS (71). Thus, reading and/or language difficulties were clinically assessed only in two of the four patients of this subgroup with right hemisphere lesions. Finally, patients R9 and R11 showed clinical deficit of executive functions.

#### 3.2.4. Abnormal Asymmetry: Ipsilesional Deficit

**Figure 5:**
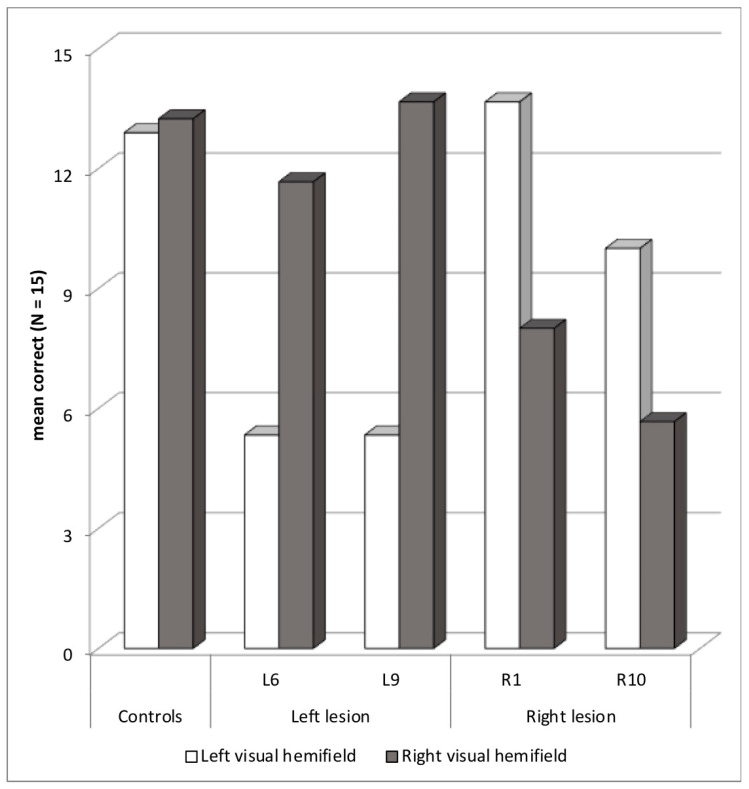
Patients with an asymmetric ipsilesional deficit: mean correct words in each visual hemifield.

Four patients showed abnormal asymmetry with a paradoxical ipsilesional deficit, two with a left hemisphere lesion and a deficit in the LVH—L6 (asymmetry *z*-score = +4.6) and L9 (+6.2)—and two with a right hemisphere lesion and a deficit in the RVH—R1 (−4.7) and R10 (−3.6) (Figure 5). Two patients, L6 and R1, also showed an abnormal selection score. In the case of L6, performance dropped in both bilateral and cued blocks compared to unilateral block. However, in the case of R1, performance was lower in the cued than in the bilateral block. At least R1 may present a mixed selection/orienting deficit. Still, no patient showed more than 50% migration errors in the cued block.

Both left-lesion patients had parietal lesions and showed strong asymmetries in favor of the ipsilesional visual hemifield in all three presentation blocks, compatible with a callosal disconnection explanation. Language was lateralized in the left hemisphere for L6, but this was not documented for L9, who was right-handed. L6 did not show signs of abnormal asymmetry with Object experiment. Disconnection may have been restricted to words in his case. Note that L6 also had a deficit in the Alouette reading test, and clinical deficit of executive functions.

In right-lesion patients, an ipsilesional deficit might be explained by a disconnection with difficulties transferring RVH words, projected to the left hemisphere, to a possible language-dominant right hemisphere. However, in fact, language was lateralized in the left hemisphere for R1 and R10, even though R10 was left-handed and R1 had difficulties with lexical access (*z*-score of −6.3 in the Lexis). In the Word experiment, R1 had low ipsilesional RVH deficit only in the bilateral and cued blocks, and R10 only in the bilateral block. An ipsilesional deficit only in some conditions of presentation is incongruent with a callosal disconnection explanation. We can propose a tentative explanation. A small deficit in the orienting of attention toward the LVH or a global slowness deficit could slow the process of identifying words in the LVH, the first to be reported in the bilateral block, leaving insufficient resources to identify and report RVH words, in R1 and R10. In the cued block, R1 may have initiated leftward orienting, as in a reading routine, producing some delay in the process of identifying words in the RVH. R1 also made four right-side omissions in the Bell cancellation test. She had been diagnosed with an epileptic right precentral lesion at a very early age: one year. She may have developed some compensatory strategies over the long term after an initial orienting deficit, but, of course, this remains speculative. 

#### 3.2.5. Other Deficits

**Figure 6:**
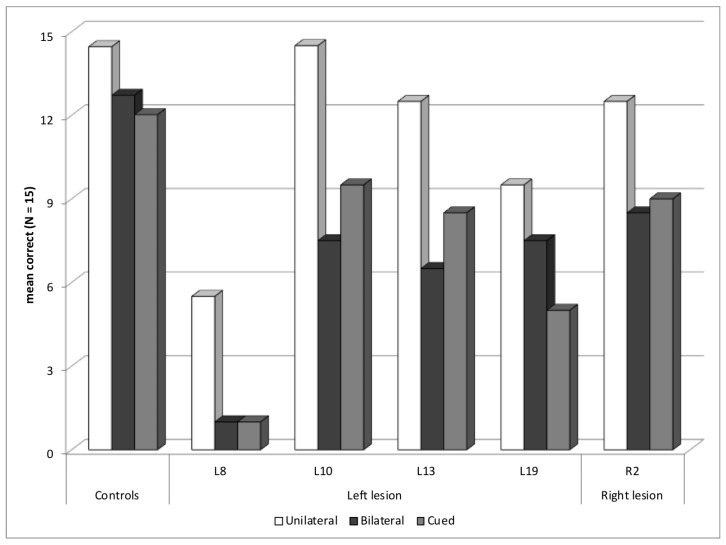
Patients with other types of deficits: mean correct words for each presentation block.

Five other patients, four with a left hemisphere lesion—L8, L10, L13, and L19—and one with a right hemisphere lesion, R2, were not included in the previous subgroups, but presented a reading deficit in the Word experiment (Figure 6). All, except L19, had abnormal reading difficulties in the bilateral block without an increased deficit in the cued block, in fact even a tendency toward improved reading in the cued block in three of them. These patients may have slow reading, so they had difficulties in identifying the two words of the bilateral block. Two patients—L8 and L10—showed signs of memory and language problems, and both had lexical access difficulties, in the Lexis test (*z*-score of −5.8, and −4.9, respectively). L8 was also abnormally slow in on the Alouette reading test, and he had a low verbal comprehension index in WAIS (63). His performance in the Word experiment was particularly low with a global *z-*score of −9.9. He also had an abnormal global score for the Object experiment, and a selection deficit for objects, without true selection deficit for words, but possibly masked by very low performance and some roof effect with words. Indeed, he showed a high rate of migration errors (57%) in the cued block, with two transposition errors and fourteen intrusion errors, but with at least five migrated letters in three of these trials. The other patients were not known to have specific reading or language difficulties.

## 4. Discussion

The main goal of this research was to investigate whether attentional processes like selecting and orienting in reading can be dissociated in adult patients with lesions or dysfunctions in the left or right hemisphere. We conducted a divided visual field study with a tachistoscopic presentation of four-letter French words in three successive presentation blocks, unilateral, bilateral, and cued, in a fixed order. A fixed order of presentation blocks was used to make direct comparisons between patients, even though a task learning effect was not controlled. However, all participants had a short practice before the experiment, to minimize the task learning effect. We made global and individual analyses, sorting performance in specific subgroups of patients according to our hypotheses.

In the global analysis, the results showed that patients with left or right epileptic focuses performed worse than control participants. The difference between patients’ and controls’ performance was found in all presentation blocks—unilateral, bilateral and cued—but was larger in the bilateral and cued blocks. However, a ceiling effect in the unilateral block, probably due to the fact that this study gave an advantage to comparable conditions by using the same presentation time in all groups, may have masked a greater deficit. In the bilateral block, patients might have had difficulties because two words had to be identified and reported, increasing the complexity of the task. In the cued block, patients might have had difficulties because attentional demand was increased even though only one word had to be reported. In the bilateral block, selection is included in a reading routine, consisting in orienting first to the leftmost then to the rightmost word, whereas selection may require more attentional resources in the cued block, in order to trigger an appropriate new schema and counteract the reading routine schema. Additionally, the perceptual load due to the cue, which was presented to the fovea simultaneously with the words, may have affected the initial stages of visual processing [50].

Individual analyses showed that 21 of the 39 patients in this study had a reading deficit with a global word score two SD below the mean score for controls in the Word experiment. This proportion of patients with reading deficits is quite high and may be explained by the severity of these patients’ epilepsy. Still, Breier et al. [31] found an almost comparable proportion of reading difficulties: 41 out of their 92 patients with intractable temporal lobe epilepsy. Only 7 of the 21 patients who had a reading deficit in our Word experiment had a speed score more than two SD below the norm for the Alouette reading test, and two had an accuracy score more than two SD below the norm. Five patients had antecedents of dyslexia in childhood and had received speech therapy. Nine patients had some oral language difficulties. This discrepancy between the Word experiment and clinical tests and evaluations may point to the greater sensitivity of reading tests using tachistoscopic presentations of words in each visual hemifield.

Concerning our main hypothesis of dissociation between selecting and orienting deficits, our study revealed that patients’ deficits may take several forms and show different aspects. A first subgroup of four patients, two with a left temporal or occipito-parietal lesion and two with a right temporal lesion, showed an abnormal selection score, calculated by the difference between unilateral and cued performance. They showed a greater reading deficit in the cued block. Selection is necessary to align the attentional window of processing with a word while other words are filtered out or ignored [4,7], and more attentional resources are needed to select the word in the cued block because a new strategy has to be applied. None of these patients had an abnormal asymmetry between LVH and RFV. Thus, these four patients may have a specific deficit in attentional selection processing, without attentional orienting deficit.

The selection deficit hypothesis in this first subgroup of patients is reinforced by the high number of intrusion errors in three of the four patients, as in patients with attentional dyslexia [9,51]. The existence of many intrusion errors in our experiment is compelling because the stimuli were not constructed to favor this type of errors. In fact, we observed two kinds of intrusion errors: true intrusion errors that involved mixing letters from both words in the display, for example reporting aire [area] instead of aide in the display aide *<* port [help, port]; and complete transposition of the word, for example clou [nail] instead of file [lane] in the display clou *>* file. We described one patient with preferential errors of the intrusion type, L15, who had a left lateral parieto-occipital ependymoma, and one patient with preferential errors of the transposition type, R13, who had a right hemisphere cortical dysplasia near the middle and inferior temporal sulci. A selection deficit with a majority of intrusion errors may correspond to difficulties ignoring and filtering letters from the non-selected stimulus and/or allocating sufficient resources to the target word. A selection deficit with a high rate of complete transposition errors may correspond to difficulties in the decision processes involved in positioning an attentional window of processing. Future studies should determine whether these reading characteristics correspond to different types of deficits and may be related to different lesion sites.

Only two of the four patients of this subgroup took the Object experiment and neither had a selection deficit for objects. Cases of attentional dyslexia are rare in the scientific literature, but the attention deficit is sometimes restricted to reading. We suggest that these patients had a reading-specific deficit affecting attentional selection because their lesion disrupted the connections between dorsal attentional and ventral word-identification areas [12]. However, another possibility is that performance improved in the Object experiment because of learning strategies.

The second subgroup of patients consisted of eight patients, four with a left and four with a right epileptic lesion mostly involving the temporal lobe, showing reading asymmetry with a contralesional deficit. None of these patients had truly detected clinical neglect but two presented isolated clinical signs of neglect. Three patients with a left hemisphere lesion and one patient with a right hemisphere lesion showed some visual field deficit, a superior quadranopia or a hemianopia, which could entirely produce or contribute to asymmetric performance. The patient with hemianopia showed a complete deficit in the RVH in the three presentation blocks—unilateral, bilateral and cued—of the Word and of the Object experiments. His asymmetric performance was most likely due to the visual field deficit. The three other patients had fewer difficulties in their contralesional visual hemifield in the unilateral presentation. Thus, in their cases, even though the quadranopia or some amblyopia may have played a role in their reading difficulties, an explanation by an attentional orienting deficit is most plausible, just as for the four other patients without visual field deficit. Note that the four patients with a right hemisphere lesion had reading difficulties in the LVH, only in the unilateral and the cued blocks but not in the bilateral block. Their LVH performance in the bilateral block was less affected by the orienting deficit, maybe because the orienting deficit was not severe and was compensated by the left report bias when two words must be reported.

Four other patients, two with a left and two with a right epileptic lesion, showed reading asymmetry with an ipsilesional deficit. This type of paradoxical asymmetry has been related to callosal disconnection, which can occur when the lesion disrupts the extension of callosal fibers within the language-dominant hemisphere [25]. Disrupted callosal pathways have been described in epilepsy, in the posterior part of the corpus callosum [52] but also in the anterior parts [53], and sometimes in relation to anti-epileptic drug therapy [54]. A callosal disconnection could explain the ipsilesional deficit in both patients with a left parietal lesion. For patients with a right lesion, the explanation is more complex. Reorganization of language dominance is not uncommon after epilepsy, and the right hemisphere may contribute to reading processes in epileptic patients [55,56]. However, a callosal disconnection cannot explain the deficit in these two right-lesion patients, as they were left-lateralized for language. It is possible that these patients, who had epilepsy for a long time, were trying to compensate for a contralesional orienting deficit. Orienting deficits are sometimes accompanied by over-compensatory strategies, especially a long time after the lesion onset [57], and with frontal lesions [58].

In these twelve patients with abnormal asymmetry, three also had an abnormal selection score, showing that they may present a mixed attentional selection/orienting deficit. However, only one had a high rate of migration errors. Also, one patient presented a high rate of migration errors without an abnormal selection score, that is without specific difficulties in the cued block, suggesting that several different attentional selection deficits may exist. Oppositely, eight patients with abnormal asymmetry did not present any sign of attentional selection deficit. To summarize, first, an asymmetric reading deficit can occur after visual field deficits, callosal disconnection or attentional deficits. Second, attentional orienting deficits can be dissociated from attention selection deficits.

Finally, the last five patients who presented reading deficits in the Word experiment, four with a left and one with a right hemisphere lesion, did not show any orienting and/or selection deficit in the Word experiment. It is out of the scope of this study, but a possibility is that these patients showed other reading, perceptual or attentional difficulties. Four of these patients had specific difficulties when two words instead of one had to be reported (bilateral block). The capacity to report word stimuli when presentation time is limited may depend on the processing speed for letters and words, thus on access to orthographic codes [59]. Similarly, a difficulty in accessing phonological codes could also slow down reading [60], and this slowness might affect the capacity to report more than one word, as in the bilateral block. Other types of deficits, for example in articulatory phonological production, could also reduce reporting speed. Thus, this subgroup of difficulties may be particularly heterogeneous.

Five patients had antecedents of dyslexia in childhood. They all showed a deficit in the Word experiment: one had a selection deficit, three had contralesional asymmetric deficits, and one had an ipsilesional asymmetric deficit. However, it is difficult to discuss the relationship between dyslexia in childhood and reading deficits in the Word experiment. First, the exact nature of the childhood dyslexia of these patients was not known, and was maybe unrelated to the deficits we found using a divided visual field paradigm. Second, the type of deficit showed by these patients in the Word experiment varied (selection, contralesional and ipsilesional asymmetrical deficits). Third, the sixteen other patients who showed a deficit in the Word experiment were not known as having dyslexia antecedent in childhood. Thus, further studies are necessary to clarify this relationship, and we recommend to use the divided visual field method more systematically in patients.

Reading deficits in the Word experiment were found in patients with right (9/18) and left (12/21) epileptic focuses. In previous studies, the side of the epileptic focus did not systematically determine the occurrence of a reading deficit [29]. Various factors that do not directly depend on the lesion site may play a role in reading deficits, such as medication, attentional disruption due to epileptic activity, and the psychosocial environment [61,62,63]. Moreover, although the linguistic processes involved in reading are normally associated with areas and connections in the left hemisphere [64,65,66], the right hemisphere may make a greater relative contribution to reading in epileptic patients than in the healthy population [55,56]. Finally, epilepsy can affect other aspects of cognition such as visuospatial processing. Of the 23 patients, only nine could be tested with object drawings, but five showed a global deficit more than two SD below the norm, raising the possibility of a more general perceptual deficit. In addition, four patients had a visual field deficit, quadranopia or hemianopia, which can affect reading. Attentional deficits may affect various capacities including reading, but they can also selectively affect reading, for example because of a disconnection between the dorsal spatial attention areas and the ventral word identification areas [64,67,68]. Another possibility is that patients with attentional reading deficits may present difficulties in accessing the frontal attention network in reading [69]. Such difficulties might explain why they cannot use partial-report cues. As for the exact site of the epileptic focus, half of our patients had a temporal focus, but the number of patients with different epileptic focus sites in the occipital, parietal and frontal lobes may be too small for a clear anatomical picture to emerge. We also noted that surgical resection was more frequent in the patients with than without a reading deficit in our Word experiment, but it is difficult to conclude that the resection itself could be the cause of the deficit, because of the small number of patients.

## 5. Conclusions

In conclusion, 21 out of 39 patients with intractable epilepsy, 12 with a left and 9 with a right hemisphere lesion, had reading deficits when tested in a divided visual field experiment. Four patients showed attentional selection deficits in reading without orienting deficit. Eight patients showed asymmetric contralesional reading deficits, caused by attentional orienting deficits in seven of them, and a visual field deficit in the last one. In six cases, the deficit was pure without selection deficit, and two patients had a mixed deficit. Other patients may present various deficits, including possible callosal disconnection with ipsilesional deficit in several patients. The dissociation of two types of spatial dyslexia, affecting selection or orienting processes, shows that the positioning of an attentional window of processing in reading may require two different spatial attention processes, selection and orienting, related to different brain networks. There were some limitations in our study, like the possible learning effects due to the blocked procedure, and to the large heterogeneity of the patients. Also, not all participants were tested with drawings of objects, so the reading specificity of the deficits was not proven. However, we believe that the presentation of words in each visual hemifield in a limited presentation time is a useful tool for detecting several reading deficits.

## Figures and Tables

**Table 1:** Number of correct words (*n* = 15) and standard deviation, in unilateral, bilateral, and cued presentation blocks, and in the left (LVH) and right (RVH) visual hemifields, for each group of participants.

	Unilateral		Bilateral		Cued	
	LVH	RVH	LVH	RVH	LVH	RVH
Controls (*n* = 66)	14.2 ± 0.9	14.9 ± 0.3	12.9 ± 1.9	12.6 ± 1.2	10.9 ± 2.1	13.7 ± 1.1
Patients with left-sided epilepsy focus (*n* = 21)	11.6 ± 3.1	12.7 ± 2.4	10.5 ± 3.5	6.9 ± 4.8	7.5 ± 4.4	9.1 ± 4.8
Patients with right-sided epilepsy focus (*n* = 18)	12.8 ± 2.3	14.3 ± 0.9	11.3 ± 2.4	8.8 ± 2.7	7.5 ± 3.4	10.2 ± 3.6

**Table 2:** Main Characteristics of Patients with a Reading Deficit in the Word Experiment.

	Age	Gender	Epilepsy Duration	Language Dominance	Lesion	Surgical Resection	Clinical Deficits	WAIS	IQ	Alouette Reading Test	
								VCI		Accuracy *z*-Score	Speed *z*-Score
Selection deficit											
L12	32	M	8	L	L anterior temporal cavernoma	Yes	language deficit, dyslexia antecedent, executive function deficit	81	83	–0.3	–3.1
L15	19	M	17	L	L occipitoparietal ependymoma	Yes	lexical access deficit, executive function deficit	75	77	–0.7	–3.2
R8	30	F	9	R	R temporal	No		94	94	0	–0.3
R13	50	M	25	L	R temporal	No		83	84	0.1	–1.9
Contralesional deficit											
L1	36	M	24	L	L anterior temporal sclerosis	Yes	right superior quadranopia, memory deficit	88	103	0.2	–1.3
L2	17	M	6	R	L opercular and insular	Yes	executive function deficit	120	102	–0.9	–0.6
L11	42	F	1	NA	L temporoparietal ganglioglioma	Yes	right hemianopia, dyslexia antecedent, lexical access deficit	NA	69	–1.5	–3.7
L17	23	M	15	R	L mesial temporal arachnoid cyst	Yes	right superior quadranopia, dyslexia antecedent, depression	90	87	–1.8	–2.1
R6	48	F	18	L	R temporoparietal, herpes encephalitis	Yes		124	105	NA	NA
R7	19	M	15	bilateral, R	R superior temporal dysplasia	No	dyslexia antecedent, lexical access deficit, verbal memory deficit	98	109	–1.1	–1.2
R9	37	M	23	L	R occipitotemporal, vascular	No	left superior quadranopia, lexical access deficit, executive function deficit	71	71	–5.3	–3.5
R11	24	F	15	L	R frontotemporal	Yes	memory deficit, executive function deficit, depression	69	87	0.2	0.3
Ipsilesional deficit											
L6	19	M	NA	L	L opercular and insular	Yes	dyslexia antecedent, executive function deficit	110	94	–3.5	–3.5
L9	29	M	20	NA	L opercular dysplasia	Yes		NA	NA	–0.5	–1.8
R1	19	F	18	L	R precentral dysplasia	Yes	lexical access deficit	112	100	0.1	–0.5
R10	20	F	7	L	R occipital dysplasia	Yes	depression	128	114	–0.1	–1.5
Other deficits											
L8	16	M	15	bilateral	L mesial temporal sclerosis	No	memory and intellectual deficits	63	66	–0.9	–3.4
L10	20	M	5	NA	L mesial temporal sclerosis	No	memory deficit	94	95	0.1	0
L13	21	F	10	bilateral	L temporal	No		NA	NA	–0.7	–1.6
L19	48	M	26	R	L temporal	No	memory deficit	84	90	0.2	–1.7
R2	16	F	13	NA	R central dysplasia	Yes	brachial hemiparesis	96	87	0.5	–0.6

Note. L = left, R = right, NA = not available, VCI = verbal comprehension index. Age and duration of epilepsy are given in years.

**Table 3:** Global, selection, and asymmetry z-scores and number of correct words and objects in left (LVH) and right (RVH) visual hemifields for each presentation block, in patients with a reading deficit in the Word experiment.

	Word Experiment						Object Experiment					
	*z*-Scores			Number Correct Words (LVH–RVH) (*n* = 15)			*z*-Scores			Number Correct Objects (LVH–RVH) (*n* = 15)		
	Global	Sel.	Asym.	Uni.	Bil.	Cued	Global	Sel.	Asym.	Uni.	Bil.	Cued
Selection deficit												
L12	−5.7	−3.0	−0.3	8–12	13–3	0–6						
L15	−8.9	−2.0	−0.3	5–8	3–3	1–1	−3.5	−1.1	−2.4	11–13	11–6	12–4
R8	−2.2	−3.4	−0.8	14–15	11–10	8–6	−1.8	−1.7	+0.1	14–13	10–12	10–7
R13	−5.0	−2.7	−0.8	9–13	8–7	7–2						
Contralesional deficit												
L1	−3.2	−0.4	−5.4	14–11	12–2	13–6						
L2	−5.8	−1.7	−2.6	10–11	8–1	7–4						
L11	−9.6	+2.0	−4.1	4–1	6–0	6–0	−7.6	+1.3	−9.3	13–0	9–0	13–0
L17	−4.7	−1.7	−6.0	13–9	12–1	10–3						
R6	−5.8	−0.4	+3.6	4–13	8–5	1–10	−8.0	+0.7	−0.2	8–4	6–5	4–6
R7	−6.0	−1.7	+2.8	6–14	8–2	0–10	−1.6	−1.1	−1.9	13–14	14–7	11–8
R9	−4.6	−2.0	+2.6	10–14	7–5	2–11						
R11	−2.7	+0.3	+3.1	9–15	10–7	5–15						
Ipsilesional deficit												
L6	−4.3	−2.4	+4.6	10–15	4–9	2–11	+0.3	−0.8	+1.0	14–15	12–14	12–10
L9	−3.3	−1.0	+6.2	9–15	4–13	3–13						
R1	−2.1	−2.0	−4.7	15–13	14–6	12–5						
R10	−4.9	+0.3	−3.6	8–10	14–1	8–6	−2.7	+0.1	−0.2	8–13	13–10	11–6
Other deficits												
L8	−9.9	−1.4	−0.5	6–5	1–1	1–1	−5.5	−2.9	+0.7	11–11	9–7	3–5
L10	−2.4	−1.7	−0.5	15–14	10–5	7–12	−1.2	−1.4	−1.3	15–13	11–11	12–7
L13	−3.6	−1.0	+1.0	12–13	10–3	3–14						
L19	−5.4	−1.9	−0.3	8–11	9–6	5–5						
R2	−2.9	−0.7	−0.3	11–14	10–7	9–9						

Note. Sel. = selection, Asym. = asymmetry, Uni. = unilateral, Bil. = bilateral.

## Data Availability

The data presented in this study are available on request from the corresponding author. The data are not publicly available due to restrictions included in the informed consent provided by participants.
